# Myoepithelial Carcinoma Ex Pleomorphic Adenoma of the Submandibular Gland: A Case Report

**DOI:** 10.7759/cureus.35722

**Published:** 2023-03-03

**Authors:** Georgia Syrnioti, Antonia Syrnioti, Alharith Abdullah, Xuehui Lui, Ernesto Mendoza

**Affiliations:** 1 Surgery, Brookdale University Hospital Medical Center, New York, USA; 2 Pathology, Aristotle University of Thessaloniki, Thessaloniki, GRC; 3 Pathology, Brookdale University Hospital Medical Center, New York, USA

**Keywords:** malignant transformation, pleomorphic adenoma, salivary gland pathology, submandibular gland surgery, submandibular gland excision, submandibular neoplasm, salivary gland tumor, carcinoma ex-pleomorphic adenoma

## Abstract

Carcinoma ex pleomorphic adenoma (Ca-ex-PA) is a rare tumor that arises from the malignant transformation of a primary or recurrent pleomorphic adenoma. Despite being benign, pleomorphic adenomas can rarely undergo malignant transformation. Risk factors include a long-standing primary tumor, a prior history of radiation exposure, increased tumor size, and recurrent disease. Ca-ex-PA usually affects patients between the sixth and eighth decades of life, approximately 10 to 20 years after the development of a pleomorphic adenoma. Patients usually present with the rapid expansion of an already existing mass. We describe a case report of a patient who presented with Ca-ex-PA of the submandibular gland. The patient underwent surgical excision of the affected gland, which was consistent with a widely invasive myoepithelial Ca-ex-PA. The patient underwent postoperative radiation to the neck and the tumor bed. No local or distant recurrence was noted during the one-year follow-up. Due to the rarity of the disease entity and the infrequent location of the tumor, this case presents a particular diagnostic and therapeutic challenge.

## Introduction

Pleomorphic adenoma is the most common tumor of the salivary glands [1]. The majority of pleomorphic adenomas arise from the parotid glands, followed by the minor salivary glands and the submandibular glands. The tumors are usually slow-growing and asymptomatic, and they most commonly exist for many years before medical attention is sought [2]. Despite their benign nature, pleomorphic adenomas can rarely undergo malignant transformation [3]. The tumor that arises from the malignant transformation of a primary or recurrent pleomorphic adenoma is termed carcinoma ex pleomorphic adenoma (Ca-ex-PA). Risk factors for the development of Ca-ex-PA include a long-standing primary tumor, a prior history of radiation exposure, increased tumor size, and recurrent disease [4]. Ca-ex-PA usually affects patients in the sixth to eighth decades of life [5]. The most commonly presented symptom is the rapid expansion of a previously existing mass. Less frequently, patients can present with pain, sensory deficits, or signs of facial nerve invasion [5]. The most common location is the parotid gland (67%), followed by the minor salivary glands (18%) and the submandibular gland (15%) [5]. We describe a case report of a patient who was diagnosed with Ca-ex-PA of the submandibular gland. Due to the rarity of the disease entity and the infrequent location of the tumor, this case presents a particular diagnostic and therapeutic challenge.

## Case presentation

**Figure 1:**
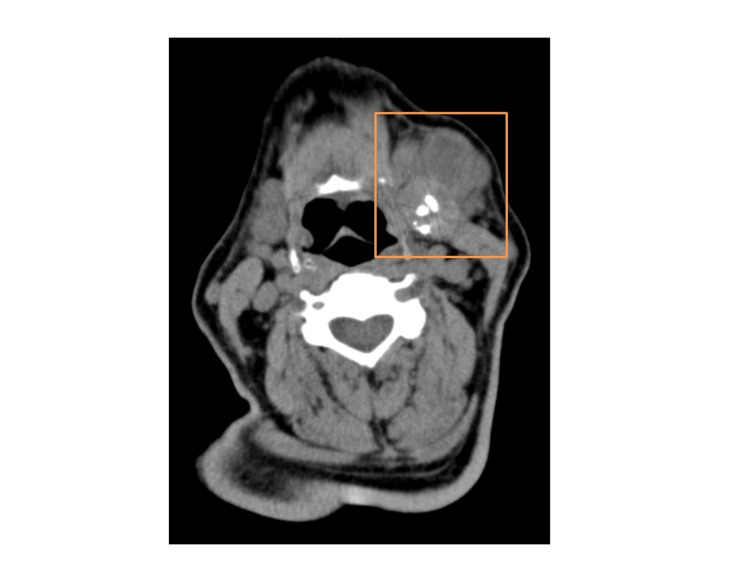
CT soft tissue neck revealing presence of a multilobulated, partially calcified left submandibular gland tumor.

An 82-year-old male presented to the head and neck surgery clinic complaining of left submandibular swelling that had rapidly increased in the last three months. The patient reported that he had a nodule in the same position for several decades but had not sought medical attention. He denied any pain, dysphagia, or difficulty breathing. He is a former smoker, and he reports that he quit smoking 20 years ago. His past medical history is significant for Parkinson’s disease, hypertension, hypothyroidism, and diabetes mellitus. He denied any personal or family history of cancer or any history of radiation exposure. Clinical examination revealed the presence of a left submandibular nodular mass, approximately 5 cm in diameter, that appeared firm and non-tender on palpation. No palpable lymph nodes could be appreciated in the neck or torso. The rest of the physical examination was unremarkable. A computed tomography of the neck was performed, which revealed a 5 cm × 4.4 cm multilobulated, partially calcified tumor of the left submandibular gland (Figure 1).

**Figure 2:**
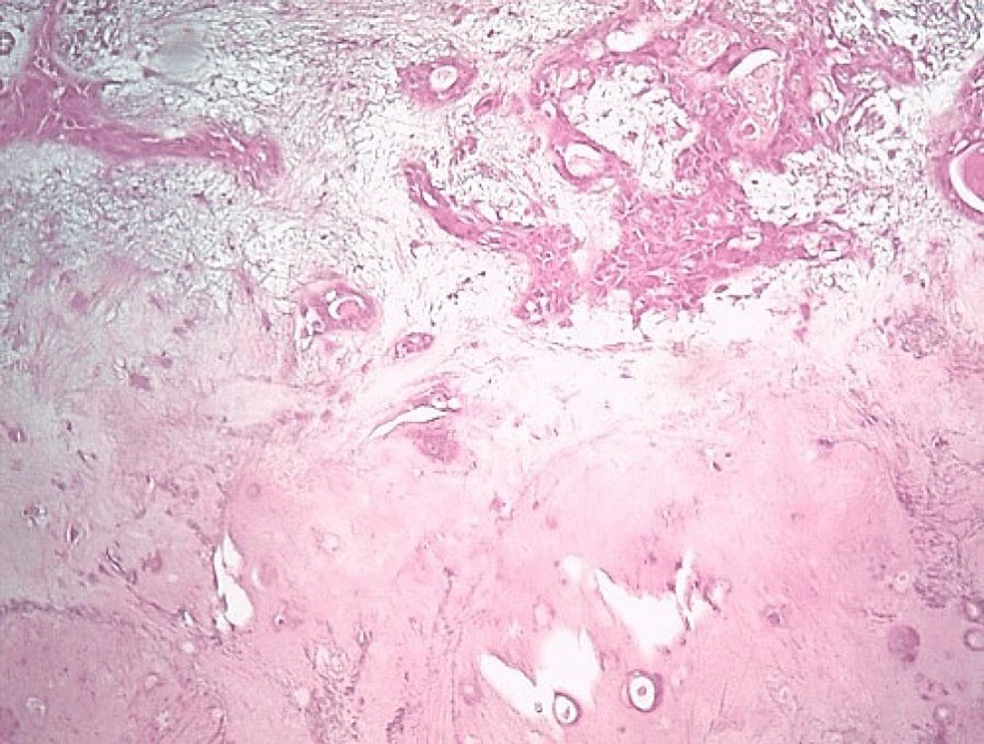
Histopathological examination of the tumor showing areas of pleomorphic adenoma (PA), consisting of epithelial, myoepithelial cells, and myxoid or chondroid stroma (hematoxylin and eosin stain, original magnification ×100).

**Figure 3:**
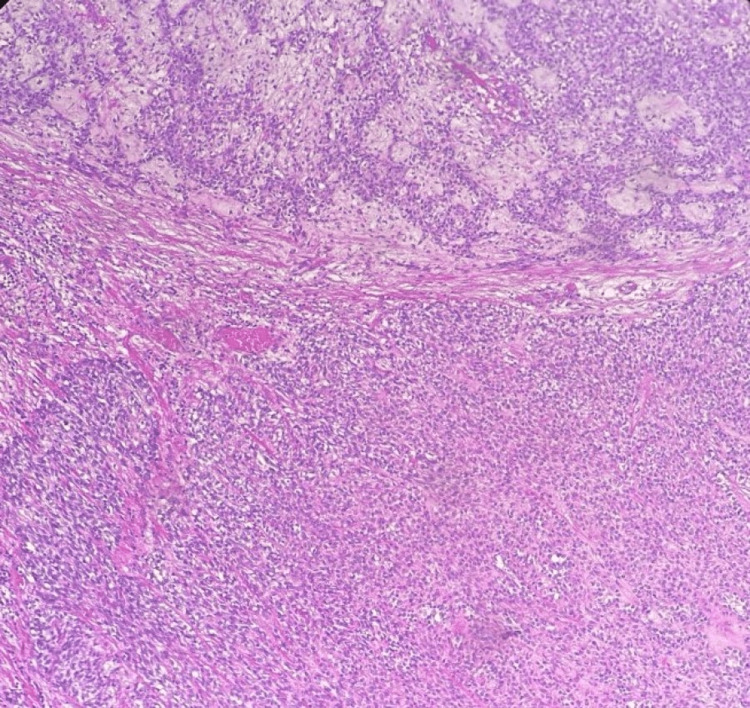
Foci of myoepithelial carcinoma demonstrating expansile invasive multinodular growth (hematoxylin and eosin, original magnification ×40).

An ultrasound-guided fine needle aspiration (FNA) of the tumor was performed. Cytology revealed few epithelial/myoepithelial cells and a scant myxoid matrix consistent with a salivary gland neoplasm. A core-needle biopsy was then pursued, which was suggestive of a myoepithelial-predominant salivary gland neoplasm with a Ki-67 proliferation index of 10-15%. Given the high likelihood of malignant transformation, the patient was taken to the operating room for left submandibular gland excision. After administration of endotracheal anesthesia, a 4 cm incision was made one inch below the left mandibular margin. The platysma muscle was incised, and after the development of superior and inferior flaps, the submandibular gland was identified. The presence of a large lobulated tumor arising from the lower portion of the gland was noted. The suspicious gland was completely excised and submitted to pathology. Histopathological examination was consistent with a widely invasive, myoepithelial carcinoma-ex-pleomorphic adenoma of the submandibular gland. The salivary gland tumor consisted of myoepithelial cells arranged in a multinodular fashion with a lobular border. The nodules were composed of a hypercellular rim with marked mitotic activity (7 mitoses/10 high-power fields), including atypical mitoses, surrounding a hypocellular center containing areas of tumor necrosis. Immunohistochemical examination confirmed the myoepithelial nature of tumor cells. Indeed, the tumor cells were positive for cytokeratins AE1/AE3 and p63. Focal positivity for calponin, smooth muscle actin (SMA), and S100 was also noted, while tumor cells were negative for CD117. No capsular invasion was noted, and the resection margins were negative for malignancy. The histopathological appearance of the tumor can be reviewed in Figures 2-3.

The patient tolerated the procedure well and was subsequently discharged. Postoperative follow-up was performed at two and four weeks, and then at three and six months after the procedure. The patient was also referred to the oncology service and received postoperative radiation to the tumor bed and neck. No local recurrence or distant metastasis was noted during one year of follow-up.

## Discussion

Salivary gland tumors are a diverse group of neoplasms with complex histopathological characteristics. Most salivary gland tumors originate in the parotid gland and less frequently in the submandibular, sublingual, and minor salivary glands. Their prevalence varies between the different populations but ranges between 3% and 10% of all head and neck neoplasms, affecting approximately 0.4-13.5 cases per 100,000 individuals every year [6]. Although the majority are benign, the risk of malignancy is higher in tumors that arise outside the parotid gland.

The most common type of salivary gland tumor is the pleomorphic adenoma, which comprises up to two-thirds of all salivary gland neoplasms [1]. It most commonly affects females in their fifth and sixth decades of life [7]. It derives its name from its mixed histopathologic appearance, which is characterized by the presence of epithelial (ductal) cells, myoepithelial cells, and chondromyxoid stroma [8]. Up to 85% of pleomorphic adenomas are located in the parotid gland, especially in the superficial lobe, followed by the minor salivary glands (10%) and submandibular glands (5%) [1]. They are usually slow-growing and asymptomatic, and they exist for many years before medical attention is sought or they are discovered incidentally on clinical examination. Pleomorphic adenomas carry a small risk of malignant transformation. The risk is higher in patients with prolonged disease (1.5% in the first 5 years, 9.5% after 15 years) [9]. Other risk factors include a history of radiation exposure, increased tumor size, and recurrent disease. For this reason, surgical excision of the affected gland is recommended once the diagnosis is established.

The malignant neoplasm that results from the transformation of a primary or recurrent pleomorphic adenoma is termed Ca-ex-PA. Other malignant forms of the pleomorphic adenoma include carcinosarcoma (a true malignant mixed tumor), metastasizing pleomorphic adenoma, and high-grade sarcoma. The Ca-ex-PA is the most common form, comprising approximately 12% of all malignant neoplasms of the salivary glands [10]. It was first described by Beahrs et al. in 1957 [11]. Ca-ex-PA usually arises in the parotid gland (67% of cases) and less frequently in the submandibular (15%) and sublingual glands (<1%) [5]. It usually affects an older age group between the sixth and eighth decades of life, approximately 10 to 20 years after the development of pleomorphic adenoma [4]. Patients usually present with the rapid expansion of an already existing mass. Depending on the location of the involved gland, facial nerve compressive symptoms, dysphagia, skin ulceration, or jaw involvement may develop. Neglected cases may also present with signs and symptoms of lymphadenopathy or metastasizing disease [5].

The mainstay of diagnosis is a cytopathological examination of the involved gland. Prior to tissue diagnosis, imaging modalities such as ultrasonography, computed tomography, and MRI can be used as adjuncts, although the ability to differentiate benign from malignant disease is limited [4]. Imaging characteristics that hint at the presence of a malignant neoplasm include irregular margins, necrosis or cystic changes, calcifications, extracapsular extension, or the presence of lymphadenopathy [12]. When malignancy is suspected, the diagnostic modality of choice is the FNA. Although it is a fast, safe, and cost-effective choice, it has limitations that are mainly attributed to sampling errors [13]. Core-needle biopsy under ultrasound guidance has been described as an accurate and safe method of diagnosing salivary gland tumors, with some studies providing significant superiority compared to FNA [14].

Pathological diagnosis relies on the presence of both malignant (carcinoma) and benign components (pleomorphic adenoma) on histologic examination. The carcinoma-adenoma ratio varies greatly between patients. Based on the histopathologic examination, the malignant component can be adenocarcinoma not otherwise specified (the most common type), adenoid cystic carcinoma, squamous cell carcinoma, myoepithelial carcinoma, or salivary duct carcinoma. Mixed types can also be encountered [15]. The extent of invasion of the carcinomatous component outside the fibrinous capsule of the affected gland can categorize Ca-ex-PA into intracapsular, minimally invasive, or widely invasive tumors. Although different cutoffs have been described in the literature in order to differentiate minimally invasive from widely invasive diseases, the WHO classification accepts the 1.5 mm distance from the capsule as the cutoff for minimally invasive tumors [8,15]. As expected, the extent of the invasion correlates with the survival and recurrence rates [15]. Intracapsular, non-invasive, and minimally invasive tumors have an excellent 5-year survival rate that approximates 100%. On the contrary, widely invasive tumors have a survival rate between 25% and 65% and can further decline to 10-35% in 15 years [16].

The prognosis is also affected by the histopathologic variant. The myoepithelial carcinoma ex pleomorphic adenoma, as reported in our case, is a rare, high-grade tumor and is associated with a poor prognosis. High expression of Ki-67, which is an index of proliferation, has also been associated with adverse outcomes [17].

The mainstay of treatment involves surgical excision of the affected gland. Since the most affected gland is the parotid, treatment usually involves parotidectomy that is either superficial in cases of intracapsular or minimally invasive tumors affecting the superficial lobe or total in cases of widely invasive carcinoma [15]. Limited evidence exists in the literature regarding indications for neck dissection. Although neck dissection is indicated in cases of clinical or radiological cervical lymph node involvement, its performance in cases of node-negative disease is currently controversial [18]. In our institution, cervical neck dissection is avoided in patients with no evidence of lymphadenopathy. Due to the rarity of the disease, limited studies have shown the effects of postoperative radiotherapy in these patients. Chen et al. reported improved local tumor control and survival benefit in patients that received postoperative radiotherapy [19], however, Shinohara et al. did not prove survival benefits in patients with the node-negative disease [20]. Although the choice for postoperative radiotherapy is individualized, more studies need to be performed to determine which patients can benefit. In our case, due to the widely invasive nature of the disease, our patient underwent postoperative radiation to the neck and tumor bed. No local recurrence or distant metastasis was noted during one year of follow-up.

## Conclusions

Pleomorphic adenoma is the most common tumor of the salivary glands. Although benign, it can rarely undergo malignant transformation. Ca-ex-PA usually develops in the parotid gland. We describe a case of Ca-ex-PA of the submandibular gland. The mainstay of management is surgical resection of the affected gland. Limited evidence exists in the literature regarding the indication of cervical neck dissection and postoperative radiation in cases of node-negative disease. Due to the widely invasive nature of the tumor, our patient underwent postoperative radiation therapy to the neck and tumor bed.

## References

[1] Almeslet AS (2020). Pleomorphic adenoma: a systematic review. Int J Clin Pediatr Dent.

[2] Piwowarczyk K, Bartkowiak E, Kosikowski P, Chou JT, Wierzbicka M (2020). Salivary gland pleomorphic adenomas presenting with extremely varied clinical courses. A single institution case-control study. Front Oncol.

[3] Valstar MH, Mast H, Ten Hove I (2021). Malignant transformation of salivary gland pleomorphic adenoma: proof of principle. J Pathol Clin Res.

[4] Kato H, Kanematsu M, Mizuta K, Ito Y, Hirose Y (2008). Carcinoma ex pleomorphic adenoma of the parotid gland: radiologic-pathologic correlation with MR imaging including diffusion-weighted imaging. AJNR Am J Neuroradiol.

[5] Antony J, Gopalan V, Smith RA, Lam AK (2012). Carcinoma ex pleomorphic adenoma: a comprehensive review of clinical, pathological and molecular data. Head Neck Pathol.

[6] Tian Z, Li L, Wang L, Hu Y, Li J (2010). Salivary gland neoplasms in oral and maxillofacial regions: a 23-year retrospective study of 6982 cases in an eastern Chinese population. Int J Oral Maxillofac Surg.

[7] Valstar MH, de Ridder M, van den Broek EC (2017). Salivary gland pleomorphic adenoma in the Netherlands: a nationwide observational study of primary tumor incidence, malignant transformation, recurrence, and risk factors for recurrence. Oral Oncol.

[8] Hernandez-Prera JC, Skálová A, Franchi A (2021). Pleomorphic adenoma: the great mimicker of malignancy. Histopathology.

[9] Malik V, Kay N, Ramsay T (2012). Pleomorphic adenoma of parotid gland in the elderly: do we always need to operate?. Arch Clin Exp Surg.

[10] Kim JW, Kwon GY, Roh JL, Choi SH, Nam SY, Kim SY, Cho KJ (2011). Carcinoma ex pleomorphic adenoma of the salivary glands: distinct clinicopathologic features and immunoprofiles between subgroups according to cellular differentiation. J Korean Med Sci.

[11] Beahrs OH, Woolner LB, Kirklin JW, Devine KD (1957). Carcinomatous transformation of mixed tumors of the parotid gland. AMA Arch Surg.

[12] Seok J, Hyun SJ, Jeong WJ, Ahn SH, Kim H, Jung YH (2019). The difference in the clinical features between carcinoma ex pleomorphic adenoma and pleomorphic adenoma. Ear Nose Throat J.

[13] Covinsky M, Cai Z, Ambelil M, Liu J, Zhu H (2018). Low grade carcinoma ex-pleomorphic adenoma : diagnosis and diagnostic challenges caused by fine needle aspiration : report of three cases and review of literature. Head Neck Pathol.

[14] Song IH, Song JS, Sung CO (2015). Accuracy of core needle biopsy versus fine needle aspiration cytology for diagnosing salivary gland tumors. J Pathol Transl Med.

[15] Khalesi S (2016). A review of carcinoma ex-pleomorphic adenoma of the salivary glands. Int J Pathol Clin Res.

[16] Neville BW, Damm DD, Allen CM, Chi AC (2019). Salivary gland pathology. Color Atlas of Oral and Maxillofacial Diseases.

[17] Sload RL, Carbone P, Johnson C, Johnson T (2016). Carcinoma ex pleomorphic adenoma of the parotid gland. Acta Otolaryngol Case Rep.

[18] Byrd S, Morris LG (2018). Neck dissection for salivary gland malignancies. Oper Tech Otolayngol Head Neck Surg.

[19] Chen AM, Garcia J, Bucci MK, Quivey JM, Eisele DW (2007). The role of postoperative radiation therapy in carcinoma ex pleomorphic adenoma of the parotid gland. Int J Radiat Oncol Biol Phys.

[20] Shinohara E, Arneson K, Perkins S (2012). The role of radiation in carcinoma ex pleomorphic adenoma. Radiat Oncol Biol.

